# When robots reshape teams: neurodynamic insights into taskwork and teamwork in search and rescue

**DOI:** 10.3389/fnrgo.2026.1771753

**Published:** 2026-02-11

**Authors:** Robert J. Spenceley, Ranjana K. Mehta

**Affiliations:** Department of Industrial and Systems Engineering, Neuroergonomics Laboratory, University of Wisconsin-Madison, Madison, WI, United States

**Keywords:** EEG, human-robot teams, hyperscanning, taskwork, teamwork

## Abstract

**Introduction:**

Beyond traditional, dyadic human-robot interaction, embedding robots into multi-human teams, such as search and rescue (SAR), requires an understanding of fundamental aspects of team composition and dynamics. While considerable work has examined how robot agents influence both taskwork and teamwork, few studies have focused on identifying which factor best explains differences in team outputs. This research investigates the neurodynamics of taskwork and teamwork as SAR teams transition between multi-human (mH) and multi-human-robot (mHR) configurations.

**Methods:**

Electroencephalogram (EEG) has been a key tool in human teamwork research because of its sensitivity to changes in cognitive states such as mental workload, sustained attention, and engagement. Specifically, EEG power spectral density (PSD), particularly frontal theta activity (4–7 Hz), has been used to assess variations in mental workload and social cognition associated with task performance. EEG hyperscanning, which evaluates interbrain synchrony between two or more individuals, using metrics such as weighted phase lag index (wPLI), has been widely employed to study teamwork among humans. In this study, PSD and EEG hyperscanning were used to analyze taskwork and teamwork in 22 teams comprising a highly engaged SAR team member (mission commander), a less-involved member (safety officer), and a navigator as they searched for victims in a virtual emergency environment. The navigator was either a trained researcher posing as a participant or a virtual robot, with the robot's performance manipulated using the Wizard of Oz technique.

**Results:**

Results for taskwork show that the social-cognitive abilities of mission commanders, but not those of safety officers, are adversely impacted by a robot navigator compared with a human navigator, despite the perceived workload remaining stable. Although team trust outcomes were similar, neural synchrony across occipital, parietal, and temporal regions increased in mHR teams relative to mH teams, indicating different neurodynamical patterns of teamwork.

**Discussion:**

The study findings provide evidence that both taskwork and teamwork are fundamentally altered in mHR teams, regardless of the effectiveness of robotic capabilities and functions, compared with mH teams. Therefore, beyond dyadic interactions, multi-human robot teaming must be viewed as a fundamentally distinct team construct rather than simply an extension of human-human teaming.

## 1 Introduction

Across domains, the introduction of autonomous agents into work may provide numerous benefits to human operators, such as reduced physical/cognitive demands, increased information processing capabilities, and improved mental models (Wright and Kaber, 2005; Tabrez et al., 2020). However, these benefits become less clear when humans are instructed to work *with* an agent in a complex environment, where outcomes may vary based on individual beliefs and experiences (e.g., trust). For example, an individual who is less trusting of robots in general will tend to interfere with the robot's activities and monitor it more frequently (Hinz et al., 2019). Importantly, for successful team performance, individual members must be supported in their task work before effective teamwork can be realized (Jonsson et al., 2021). *Taskwork*, which relates to individual abilities and characteristics (e.g., executive functioning, cognitive control, working memory), has been linked to individual task performance and error management (Salas et al., 2005). On the other hand, *teamwork*, referring to how well an agent coordinates, cooperates, and collaborates with another agent, has been linked to collective performance (Cooke et al., 2013). Autonomous partners are generally constrained in their ability to support flexible performance and may be unable to support both taskwork and teamwork in a multi-human team (Demir et al., 2019). Thus, there is an apparent need to understand which team components are fundamentally altered by the inclusion of an autonomous agent to improve the design of future teams.

As autonomous agents become more accessible through the rise of artificial intelligence (AI), there is an apparent need to understand how humans may work with AI agents in fluid, collaborative settings. The impact of autonomous/AI partners on multi-human teams is speculated to be positive, particularly in situations where the autonomous system has a complementary skill set to human members (Gromek and Lowe, 2025). However, much of the existing literature on such teams has focused solely on dyadic interaction, in which one human works with an AI agent, which may not exhibit the same teaming dynamics as a larger, heterogeneous group. Interestingly, some recently published studies with multi-human-AI teams suggest that teams perform worse than all human teams and exhibit reduced inter-team communication (Gromek and Lowe, 2025).

### 1.1 Robots in search and rescue

The field of disaster robotics has developed several robots that support first responders by reducing the physical demands of their work [e.g., heavy lifting (Zhang et al., 2025)] and alleviating cognitive load [e.g., by handling navigation, allowing responders to focus on their specific goals (Su et al., 2014)]. One particularly relevant domain, search and rescue (SAR), is a crucial part of disaster response, focused on rescuing trapped victims in a timely manner. In SAR, responders are constrained by spatial demands (e.g., unfamiliar environments and tight spaces), dynamic environments, and limited ability to complete tasks in situ, necessitating close teamwork to accomplish goals (Bearman et al., 2023). In such situations, intragroup features, such as collaboration, communication, cooperation, and shared mental models, become crucial for the safety of responders and civilians (Kleiner et al., 2005). To support these features, team members must develop a shared knowledge base, which requires coordination of each member's past activities, completed tasks, and remaining objectives (Han et al., 2024). While a large body of research exists to explain human-robot interactions in a dyadic team configuration (single robot, single human), few studies have aimed to examine how these robots integrate into a fluid team (comprising more than one human), in a dynamic environment where traditional concerns (such as trust, dependence, and safety) may be less applicable, as the majority of cognitive resources are expended on completing taskwork (Lyons et al., 2017; Cavicchi et al., 2025).

### 1.2 Taskwork vs. teamwork

From a taskwork perspective, a reliable agent, such as a robot, can help manage cognitive load by completing lower-level tasks, thereby freeing a human teammate to focus on complex components (Chen and Barnes, 2014). Many benefits may accrue at the individual level by adding an autonomous agent, such as reducing mental workload (Parasuraman and Wickens, 2008; Kaber and Endsley, 1997) and maintaining situation awareness (O'Neil et al., 2020). For autonomous agents to be adopted into human-autonomy teams (HATs) and human-robot teams (HRTs), they must achieve higher levels of automation, enabling them to act and make decisions independently of human agents (McNeese et al., 2018). Namely, HATs/HRTs are consistently reported to be less efficient than all human teams at information processing, situation awareness, and team performance (Gromek and Lowe, 2025; Kleiner et al., 2005). In addition, the theory of Interactive Team Cognition asserts that communication and coordination among teams are the lifeblood of effective teamwork (Ho et al., 2024). Robots have been found to successfully facilitate communication and collaboration in various contexts, including classrooms (Ouyang and Xu, 2024; Taylor et al., 2024), healthcare settings (Weerarathna et al., 2023; Lim et al., 2024), and manufacturing environments (Ferrari and Secchi, 2024; Scholl, 1996). Interestingly, many of these teamwork benefits are attributed to taskwork benefits (i.e., reduced workload), which provide limited insight into *how* an autonomous agent benefits a *team*.

Many previous models of teamwork share a common theme: a focus on social interactions, including items such as team chemistry, connectedness, shared mental models, and a supportive outlook (Johnson, 2021; Schmutz et al., 2019). Social factors of teamwork have been positively associated with context-specific outcomes, including clinical performance, reduced search time in SAR tasks, and collaboration (Rahman et al., 2018; Ellwart and Antoni, 2017). Each of these concepts falls under the umbrella of team cognition, referring to how knowledge is distributed among members (Majchrzak et al., 2007). Strong team cognition may be a factor in team resilience, as it enables teams to respond more effectively to unexpected events, distribute resources, and allocate tasks (McNeese et al., 2016). Several additional studies have identified shared mental models (SMM) as a fundamental explanation of team processes (Manges et al., 2020; Andrews et al., 2023). In dynamic environments, such as SAR, shared mental models become increasingly important because they are essential for understanding and predicting team members' behaviors (Atweh et al., 2022). However, many components of dynamic teamwork remain relatively understudied in the HRT literature.

Although the previously outlined concepts remain crucial to the team's success, individual-level abilities (i.e., task work) should not be overlooked. Several cognitive capabilities (e.g., working memory, executive functions, spatial awareness, and emotion regulation) have been identified as essential for team members to collaborate and achieve goals (Los et al., 2020). A particularly important division of these skills, namely executive functioning, has numerous connections to positive team outcomes, including working memory, cognitive flexibility, cognitive control, and inhibition (Los et al., 2020; Hart and Staveland, 1988). Executive function abilities are cognitively demanding skills that are significantly affected by high cognitive load, fatigue, and stress (Manges et al., 2020).

### 1.3 Measuring taskwork and teamwork

Several measures have been developed to assess individual states and performance [i.e., for *taskwork;* NASA Task Load Index (TLX) (Broadbent et al., 1982), Cognitive Failures Questionnaire (Vallat-Azouvi et al., 2012), Working Memory Questionnaire (Porter, 2010)]. While they may be useful for establishing a baseline of individual workload or performance, using them to make major decisions about team composition and resource allocation may be inadequate (Saby and Marshall, 2013).

To objectively quantify taskwork, electroencephalogram (EEG) power spectral density (PSD) can be evaluated, decomposing neural activity into five distinct frequency bands (Chikhi et al., 2022): delta (0.5–4 HZ), theta (4–7 Hz), alpha (8–12 Hz), beta (13–30 Hz), and gamma (30–100 Hz). With PSD, both the absolute and proportional (relative) power of each band may be attributed to various cognitive states like mental workload and social cognition (Saxe and Kanwisher, 2003). Several brain regions have been identified as important for team activities. The temporo-parietal junction (TPJ) has been implicated in aspects of social cognition, including theory of mind, cognitive flexibility, and cooperative decision-making (Tei et al., 2017, 2021; Tan et al., 2024; Wang et al., 2016). Cognitive components such as these will play a large role in the social interactions underlying teaming behavior and may be largely responsible for collective performance. Frontal regions of the brain [e.g., the prefrontal cortex (PFC)] are associated with executive functions such as working memory, cognitive control, and attention, which may be important for team members to perform their individual roles and manage dynamic task demands (Wang et al., 2016). In both the PFC and TPJ, theta PSD has been repeatedly used to study workload and social cognition (Cavanagh and Frank, 2014; Wang et al., 2016; Schiller et al., 2019). In a SAR environment, where even a few seconds can be the difference between life and death, identifying inefficiencies in team neurodynamics and developing solutions are critical.

Components of teamwork have been extensively quantified using behavioral and subjective measures (Ma et al., 2022). For example, several scales quantify teamwork using collaborative metrics [Collaborative Assessment Survey (CAS) (Vasodavan et al., 2022), Interprofessional Team Collaboration Scale (Orchard et al., 2012)], team efficiency (which consists mainly of custom scales that quantify context-dependent features of teamwork) (Schmutz et al., 2019), or affective measures [Team Strategies and Tools to Enhance Performance and Patient Safety (TeamSTEPPS) (King et al., 2008), Group Environment Questionnaire (Carron et al., 1985)]. One particularly interesting scale, proposed by (Costa and Anderson, 2011), encompasses many of these factors while predicting team performance, collaboration, and team efficiency, all important features in a SAR environment. Additionally, many teamwork assessments rely on video observation [e.g., the Assessment of Obstetric Team Performance (Toyama et al., 2011) and the leadership and team behavior measurement tool (Nothhelfer et al., 2023)], which may be subject to researcher bias and error. Importantly, each of these scales, and many other custom scales not mentioned here, measures teamwork in unique ways, which may impact the generalizability of results.

Fewer studies have attempted to integrate neuroimaging data to understand the cognitive components implicated in teamwork. EEG hyperscanning appears to be a particularly promising method for this domain, given its high temporal resolution, which enables the identification and evaluation of neural markers of teamwork (Czeszumski et al., 2020). With hyperscanning, researchers can simultaneously record the neural activity of multiple subjects to compare activity during time-locked events (Léné et al., 2021). In the present work, we use EEG hyperscanning to evaluate teamwork by analyzing frequency bands at electrode sites or inter-brain synchrony measures. In the following section, we outline the components relevant to the current body of work.

Historically, popular metrics of teamwork have relied on performance outcomes and subjective measures, which, while providing insights into specific components of teamwork, may be insufficient to capture dynamic interactions over extended periods (Demir et al., 2019; Ma et al., 2022). EEG hyperscanning involves evaluating these frequency bands across multiple brains at synchronized time points. Hyperscanning provides the unique benefit of offering continuous, objective insights into team dynamics that cannot be captured by other metrics. For example, cortical synchrony between the frontal and prefrontal regions of the brain has been linked to collaboration within teams (Léné et al., 2021). Similarly, inter-brain synchrony (IBS) is significantly more prevalent between cooperating groups than between competing groups, suggesting that synchrony may begin when a partner's actions are recognized (Reinero et al., 2020). It has been proposed that IBS extends beyond shared experiences and could serve as an indicator of social cohesion among team members (Liu et al., 2021). Additionally, higher cognitive load may impair information processing, reducing team members' ability to detect social cues that contribute to IBS (Hayati et al., 2025). Finally, several studies (Astolfi et al., 2020; Pan et al., 2020) have reported that higher IBS is positively associated with team performance outcomes.

Some studies have suggested that certain forms of synchrony may be maladaptive, a phenomenon referred to here as over-synchronization. This argument has been more clearly outlined in the coordination vs. competition literature, where teams that collaborate well elicit a functional form of neural synchrony associated with peaks in performance, whereas those that compete elicit less (Kayhan et al., 2022). Namely, functional types of synchronies typically appear alongside fluid communication, collaboration, and social cohesion (Léné et al., 2021; Reinero et al., 2020; Liu et al., 2021). On the other hand, it may be possible for teams to over-synchronize on undesirable qualities, such as shared distractions, in less coordinated teams (Reinero et al., 2021). Over-synchronization is particularly relevant when discussing autonomous and robotic teaming, as operator trust in agents has been found to be associated with monitoring, compliance, and reliance with/on autonomous partners (Lee and See, 2004). Under this interpretation, a lack of trust from one team member could lead to reduced trust from the other via IBS. The distinction between functional and over-synchrony could be a promising direction for investigating why some hyperscanning studies find that neural synchrony predicts better performance, whereas others do not.

Hyperscanning studies typically quantify neural synchrony using coherence measures, such as total interdependence, correlation-based measures, such as cross-correlation and inter-brain correlation, or phase measures, such as phase-locking values (PLV) and phase lag index (PLI) (Kayhan et al., 2022; Hakim et al., 2023; Valencia and Froese, 2020). Coherence and correlation measures are widely used in hyperscanning studies for their ability to reveal relationships between two sites (Hakim et al., 2023). Both measures can be used with a high degree of success when the goal is to understand how individuals respond to the same stimulus, coordinate on tasks requiring social and motor cooperation, and (Nolte et al., 2004; Valencia and Froese, 2020). While hyperscanning appears to be a promising method, several studies have identified potential pitfalls, which should be considered when interpreting results. Namely, synchrony measures may be better suited for identifying the cognitive components that contribute to coordination rather than predicting team performance, which has been more accurately predicted using other forms of physiological data (Qin Y. et al., 2025). Additionally, coherence and correlation-based measures both suffer from the common source problem (which assumes that one source of neural activity will be detected across multiple electrodes), capturing inflated zero-lag synchrony that may be more closely related to co-activation than neural coupling (Kayhan et al., 2022; Bastos and Schoffelen, 2016; Valencia and Froese, 2020).

Phase measures, on the other hand, have been successfully used in teaming situations where stimuli reflect natural teamwork and are more likely to signify true synchrony (Pan et al., 2020). While PLV and PLI are similar in principle, measuring the similarity between the cortical oscillations of certain frequency bands between two brains, PLI is viewed as a robust measure, as it does not suffer from the common source problem. PLI has been used to identify coordinated behavior, verbal interactions, and leader-follower relationships in human teams (Sänger et al., 2012, 2013; Short et al., 2021; Lyons et al., 2017). However, PLI is sensitive to noise, and thus, the weighted phase lag index (wPLI) was developed. The main benefit of the wPLI is its ability to reduce the effects of volume conduction and noise while accounting for the lag between the 2 signals (Shafiei et al., 2024; Czeszumski et al., 2020; Abubshait and Wiese, 2022). To reduce the likelihood of capturing “false” synchrony, and given its success in similar teaming settings, wPLI was chosen as the synchrony measure for this study. For a comprehensive review of hyperscanning methodologies, see (Bastos and Schoffelen, 2016).

### 1.4 Gaps and hypotheses

Despite extensive research on behavioral and subjective outcomes in human-robot teaming, relatively little is known about the neurocognitive mechanisms that drive teamwork in dynamic settings. *Are they similar to those of all human teams?* While prior EEG studies have quantified how individual features of taskwork (e.g., workload, social cognition) and teamwork (e.g., IBS) affect teams, few have examined how these dimensions interact to influence teams as a whole. In the present study, we aim to investigate the viability of robots as *teammates* in a SAR environment by (1) determining the role-specific changes in neural signatures of taskwork in all human (mH) and human-robot (mHR) teams, and (2) identifying differences in neurodynamic signatures of teamwork across all human and human-robot teams. To investigate these aims, we propose four hypotheses:

- *Hypothesis 1.1:* We expect that, when compared to mH teams, mHR team configuration will significantly reduce the cognitive load of highly involved team members (mission commander), enabling better cognitive control, measured with Theta PSD at frontal sites.
- *Hypothesis 1*.2: We expect that, compared with mH teams, the mHR team configuration will alter social cognition among team members, as measured by TPJ theta activity.
- *Hypothesis 2.1:* We expect that the neurodynamic signatures of teams, measured using wPLI, will differ between team compositions (mH vs. mHR).
- *Hypothesis 2.2:* Neural synchrony will predict mission performance, measured using the number of victims rescued, more accurately than team trust measures.

## 2 Methods

To evaluate the study aims, participants were recruited to complete a virtual SAR task in multi-human (mH) and multi-human-robot (mHR) team configurations. Team members were assigned interdependent roles, and several metrics of teamwork and task performance were evaluated. Participants were randomly assigned to the “Mission Commander” or “Safety Officer” role and completed all experimental trials under their assigned role. An additional role, the navigator, was either human or robot, with equal performance. This study was approved by the University Institutional Review Board at Texas A&M University (IRB20220473DCR).

### 2.1 Participants

42 participants were recruited for the study, forming 21 teams. Overall, the study included 19 females and 23 males, divided into four female-female teams, seven male-male teams, and 11 male-female teams. Participants were mainly younger adults, fluent in English, with a mean age of 24.14 ± 3.05 years, who spent an average of 4.75 ± 5.55 h per week playing video games. Before the experimental session began, informed consent was obtained from all participants, and members of the research team confirmed that participants had not previously met. Subsequently, the two participants, along with a confederate who was a member of the research team (unknown to the participants), completed a brief team-familiarization task. The task, lasting ~5–10 min, required participants to introduce themselves to the team and plan a picnic together. This task was adapted from (Javaid and Estivill-Castro, 2021) and is an integral step in ensuring a baseline level of team familiarization.

### 2.2 Experimental protocol and teaming configurations

#### 2.2.1 Experimental protocol

**Figure 1:**
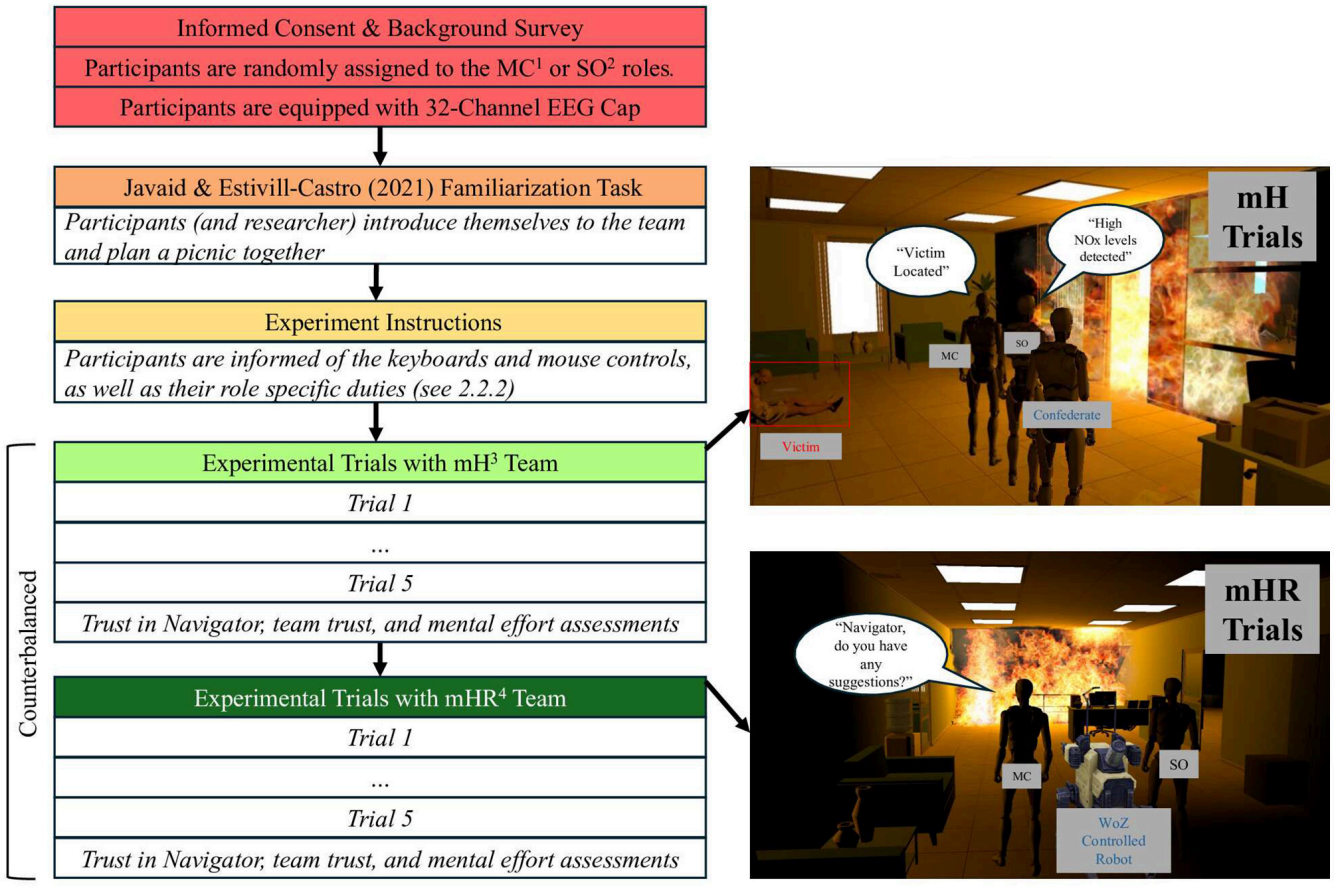
Experimental protocol. The experiment employed a within-subjects design, in which all participants completed trials under each team condition. Participants remained in their assigned role for the duration of the study. 1. Mission Commanders; 2. Safety Officers; 3. Multi-human; 4. Multi-human robot. A realistic search and rescue environment was created in Unity, where participants completed trials under both multi-human (mH), and multi-human-robot (mHR) teams.

A realistic 3D environment was created in Unity, consisting of 2 identical floors that simulated a SAR environment. The building environment included 16 victims in randomized locations and several “unsafe” areas. These areas included hot spots, where large flames engulfed a portion of a room, producing an unsafe level of nitrogen dioxide. To navigate the scene, participants used a keyboard-and-mouse configuration, moving their character with the arrow keys and panning the camera with the mouse. Participants were given sufficient time to familiarize themselves with the testbed controls, the tasks, and their roles. Participants underwent five search trials across both mH and mHR team conditions, each lasting 3 min. Participants were instructed to log as many victims as possible within each trial. The order of team configurations was counterbalanced across experimental sessions to the best of our ability, with nine teams completing mH trials first and 12 completing mHR trials first. Additional details of the experimental protocol are shown in Figure 1.

#### 2.2.2 Team configurations

**Figure 2:**
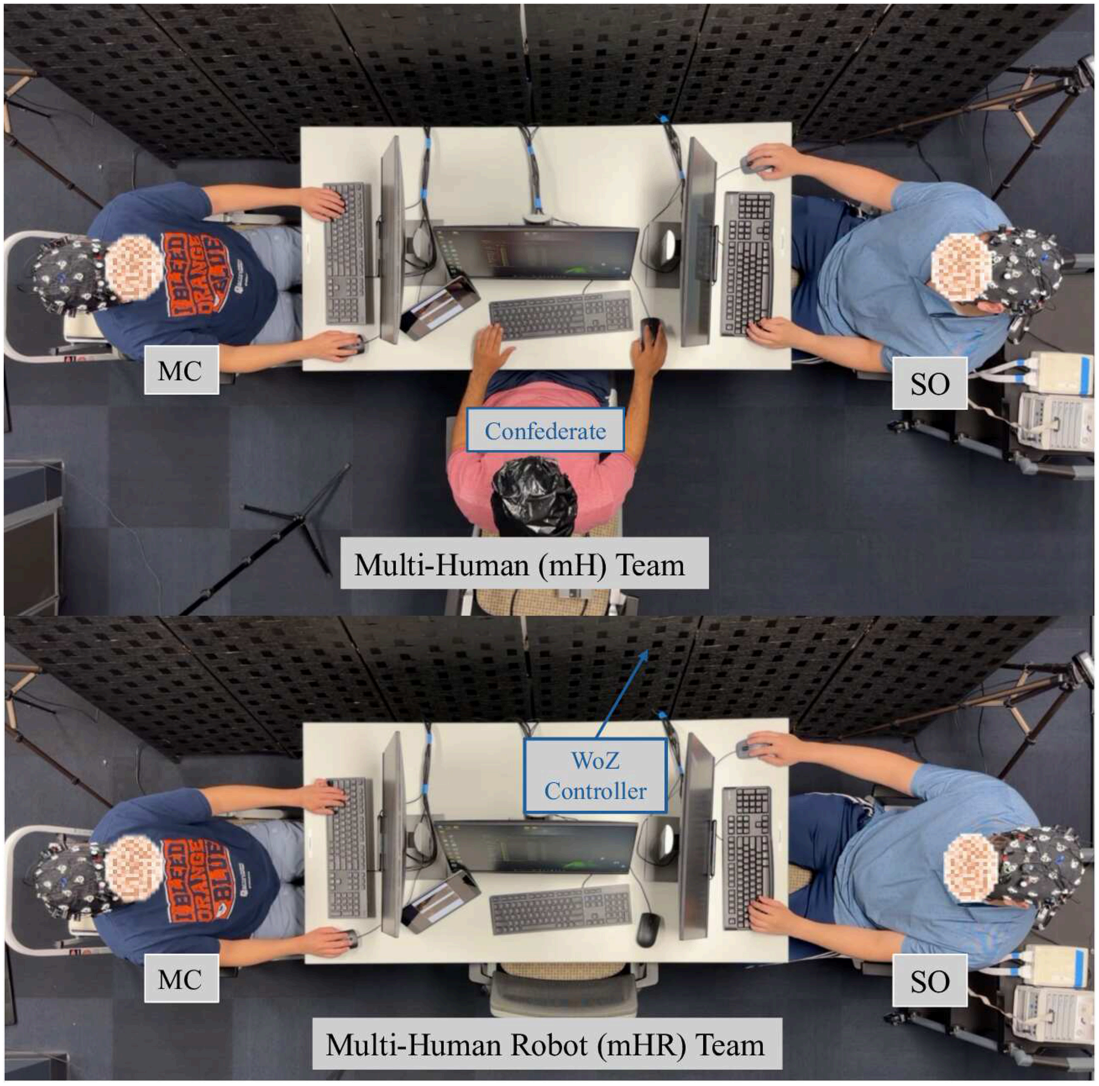
Team configurations. Participants in this study experienced trials under 2 team configurations. mH teams **(top)** consisted of the 2 participants and a trained researcher. Participants were randomly assigned to either the mission commander (MC) or safety officer (SO) role. mHR teams **(bottom)** included a robot with comparable capabilities, which was controlled with the Wizard of Oz method. During Wizard of Oz (WoZ) control, researchers left their original position with the team and controlled the robot from a computer stationed behind a curtain.

Participants were randomly assigned to 1 of 2 roles: mission commanders (MC) or safety officers (SO), each with interdependent responsibilities (Figure 2). The mission commander was responsible for collaborating with the navigator and the safety officer, making decisions, leading the team, and documenting the locations of any victims. The safety officer was responsible for monitoring nitrous oxide (NOx) levels (displayed as low, medium, or high) solely based on the output on their terminal, and for determining whether an area was safe for the team to enter. Lastly, the navigator, who was either a confederate (in the mH condition) or a robot (in the mHR condition), was responsible for directing the team to victims but provided information only when explicitly asked by the MC or SO using the prompt: “*Navigator, do you have any suggestions?*” Across both the mH and mHR conditions, the navigator would provide the same prompts, ensuring the same information was made available to the teams. Equal performance in the mHR condition was achieved using the *Wizard of Oz* method, where an experimenter controlled the robot and gave instructions from a pre-recorded list of commands (i.e., “I suggest taking a U-turn at the next intersection.”). All communications between team members were conducted verbally.

### 2.3 Measurements

#### 2.3.1 Electroencephalogram

Upon arrival at the lab, both participants were fitted with BrainVision 32-electrode EEG caps using the 10–20 placement method (Jasper, 1958; Homan et al., 1987). Data were collected using two separate Brain Vision systems (two amplifiers and two caps, one per team member). High-viscosity electrode gel was applied to each of the 32 electrodes, ensuring that the impedance of each electrode was at or below 35 kΩ (Luck, 2014). Although traditional passive EEG recording systems suggest that impedance levels should be kept at or below 5–10 kΩ, the BrainVision systems used are considered active systems (which have small amplifiers built into the electrode site) and can detect signals with impedance up to 500 kΩ (Brain Products GmbH). EEG systems were used to evaluate the neurodynamic responses of the team and individual performance. Neural activity was simultaneously recorded for both participants throughout the task. Although data were not collected from the confederate, the experimenters instrumented the confederate with dummy equipment similar to that used by the two participants.

To process EEG data, MATLAB version R2024b was used with the EEGLAB [v2024.0; (Delorme and Makeig, 2004)], ERPLAB [v10.1; (Lopez-Calderon and Luck, 2014)], and the Signal Processing Toolbox (v24.2) plugins. A comprehensive review of EEG preprocessing methods is published elsewhere (Luck, 2014; Bigdely-Shamlo et al., 2015). The preprocessing steps employed in this study are presented briefly below. EEG preprocessing started with the removal of the Direct Current (DC) offset and manual filtering of the data. DC offset refers to unwanted, and often systematic, voltage additions to the EEG recording that are not neural activity (i.e., electrode “noise” or artifacts). Manual filtering included removing noisy channels/segments. Note that no data was deleted during a trial. Next, a 0.1–30 Hz IIR Butterworth filter was applied to reduce the impact of non-neural artifacts, such as ocular activity. Independent Component Analysis (ICA) was performed to identify and remove channels with high ocular activity and to reduce noise from blinks, using an Independent Component Label (ICLabel) and an automated plugin that classifies EEG data (i.e., brain, eye, channel noise, etc.) (Makeig et al., 1995). ICLabel and ICA can identify known neural artifacts in EEG data (Jung et al., 2000). Lastly, the data were re-referenced using a common average reference across all sites (Luck, 2014).

Once the data were preprocessed, additional analyses were performed to calculate the Power Spectral Density (PSD) and the weighted Phase Lag Index (wPLI). Each method was selected for its ability to quantify individual and team neurodynamics over time. PSD is calculated as:


$$\begin{array}{l} \hat{S_{x}^{W}}\left(\omega_{k}\right)\equiv \frac{1}{K}\overset{K-1}{\underset{m=0}{\sum }}P_{x_{m},M}\left(\omega_{k}\right). \end{array}$$ (1)

where *SxW(*ω*k)* denotes the PSD estimate at frequency ω*k*, obtained by averaging across K windowed segments of length M, where *Px*_*m*1_*M(*ω*k)* is the modified periodogram of the *m*^*th*^ segment (Welch, 1967).

wPLI is calculated as:


$$\begin{array}{l} \Phi =\frac{\left|E\left\{ℑ\left\{X\right\}\right\}\right|}{E\left\{\left|ℑ\left\{X\right\}\right|\right\}}\;=\;\frac{\left|E\left\{ℑ\left\{X\right\}\right\}\text{sgn}\left(ℑ\left\{X\right\}\right)\right|}{E\left\{\left|ℑ\left\{X\right\}\right|\right\}} \end{array}$$ (2)

where Φ is the weighted Phase Lag Index value, X is the cross-spectrum between two signals, I{X} is its imaginary part, |I{X}| is the magnitude of this imaginary component, sgn(I{X}) indicates whether one signal leads or lags, E{·} denotes the expectation across time or trials, and Pr{·} represents probability.

To calculate PSD (Equation 1) and wPLI (Equation 2), the data were first partitioned into 180-s segments corresponding to each trial. Next, frequency bands were defined as Delta (0.5–4 Hz), Theta (4–7 Hz), Beta (8–12 Hz), and Alpha (13–30 Hz) (Klimesch, 1999; Niedermeyer and da Silva, 2004; Harmony, 2013). Absolute and relative PSD of each frequency band were then extracted from all segments using a 1-s sliding window with a 0.5-s Welch overlap, and Welch's method (Bruns, 2004; Vinck et al., 2011). wPLI was calculated using the same segmentation, using a bandpass filter and a Hilbert transform to extract instantaneous phase (Vinck et al., 2011). wPLI was calculated for each band and as a broadband value (0.1–30 Hz). PSD is reported on a continuous scale, whereas wPLI is reported as a proportion ranging from 0 to 1, with 1 indicating the highest degree of synchrony (Vinck et al., 2011).

#### 2.3.2 Subjective and behavioral measures

Demographic information, including age, sex, and video game experience, was collected via a survey at the start of the experiment. After each trial, participants answered a subset of questions from (Lyons et al., 2017), on a 7-point Likert scale assessing trust in the navigator (“*I trust the navigator*”; 1 = “Completely Disagree”, 7 = “Completely Agree”), team trust, which consisted of 6 of the 21 questions, (“*I trust my team*”; 1 = “Completely Disagree 7 = “Completely Agree”), and mental effort (“*What is your mental effort?*” 1 = “Low” 7 = “High”). During each trial, the number of victims rescued was recorded by comparing the participants “in-game” X, Y coordinates with the known, victim coordinates. When the coordinates matched, the victim was logged as saved.

Additional behavioral metrics included the number of times the MC asked the navigator for directions, the number of times the SO provided hazard communication status, and the duration of time the team stopped communicating (stopped communication). Video recordings were made of each experimental session, and researchers manually counted each instance of these measures (i.e., “Should we follow the navigator?” Or “High hazard in that room”). The number of times the MC asked the navigator for directions was used as a proxy for trust, with more requests from the navigator indicating lower trust (Sapp et al., 2019). Similarly, the number of times the SO provided hazard communication served as a proxy for engagement levels, as less involved members should report fewer hazards (Luo et al., 2025). Each instance of a participant communicating about following the navigator and hazards was summed for each trial and averaged across team configurations. Lastly, communication was measured as an additional indicator of team efficiency, with more frequent interruptions reflecting team confusion or increased monitoring or partner involvement (Lasota and Shah, 2015).

### 2.4 Statistical analyses

The first step of statistical analysis was to identify and remove outliers. A threshold of z = 3 was set, and any point exceeding it was removed from the dataset. Next, the windows were collapsed across trials for each participant to the median level. For each trial, a single value was calculated for all frequency bands and for the broadband average (0.5–30 Hz). Medians were used rather than means to reduce the influence of noisy points that persisted through processing. Next, the data were tested for normality using the Shapiro-Wilk test. The subjective measures, namely the mental effort, team trust, and trust in the navigator, were normally distributed, while all other measures were non-normal. Theta PSD [*Hypothesis 1.1* (frontal) and *Hypothesis 1.2* (temporo-parietal)] and wPLI (*Hypothesis 2.1* [at each frequency band and broadband)] values were tested for team configuration difference (mH vs. mHR) using a paired Wilcoxon signed-rank test separately for MC and SO. Performance (i.e., number of victims), trust, and workload ratings (i.e., trust in the navigator, team trust, workload) were compared across team configurations using a paired *t*-test. To assess whether neural synchrony and team trust predict mission performance (*Hypothesis 2.2*), a multiple linear regression was conducted with broadband global wPLI and team trust as predictor variables and the number of victims as the dependent variable. Lastly, the times the MC asked to follow the navigator, hazards communication counts, and total stopped communication were compared using a paired *t*-test. Statistical significance was assessed at an alpha = 0.05. Given that the goal of the present study is to explore potential differences, uncorrected *p*-values are reported in the results section.

## 3 Results

### 3.1 Trust, workload, and performance

**Figure 3:**
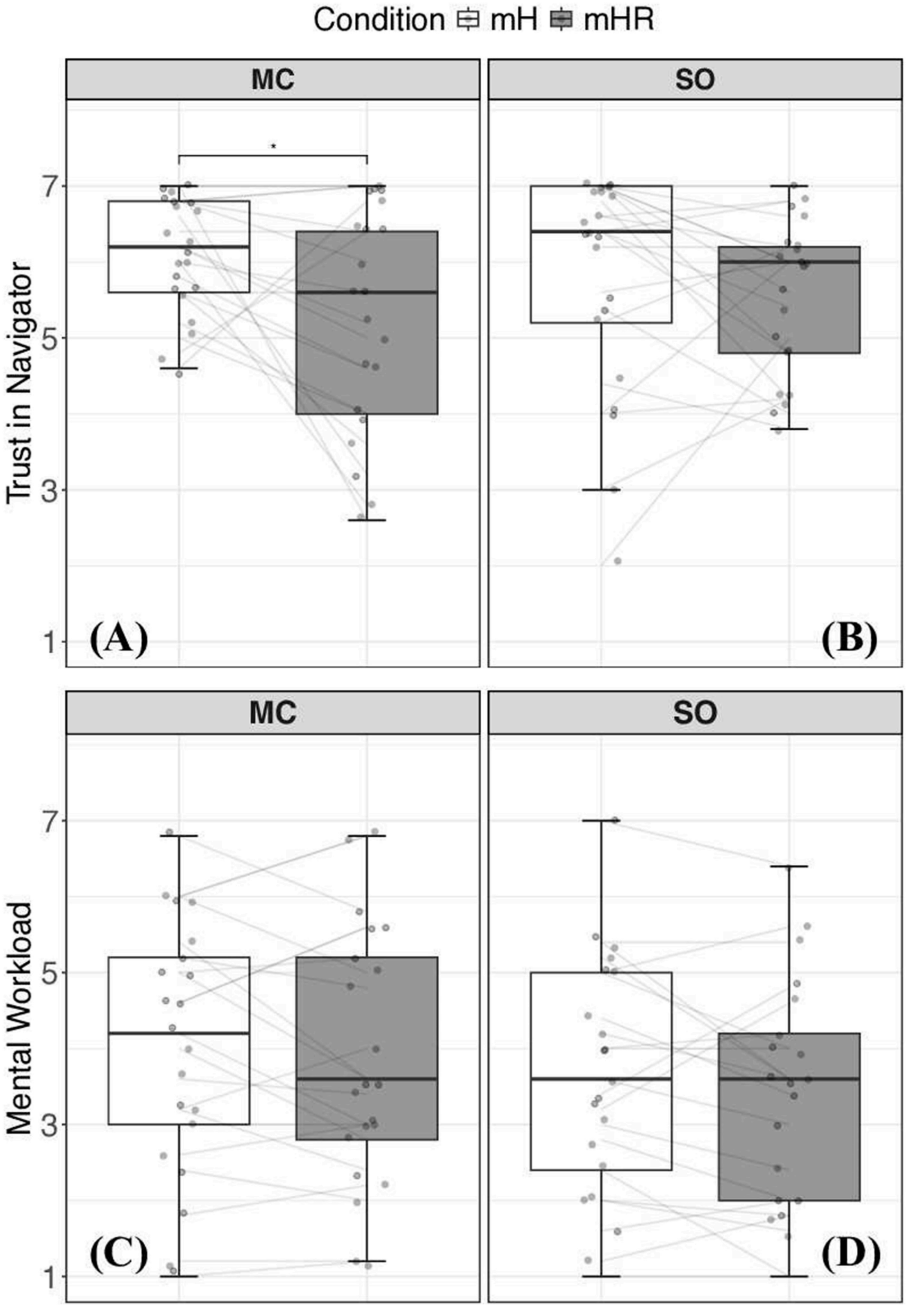
Trust in the navigator **(A, B)** and perceived workload **(C, D)** for mission commanders (MC; left) and safety officers (SO; right) under Multi-Human (mH) and Multi-Human Robot (mHR) team configuration. *Represents significant differences (*p* < 0.05) between team conditions. Significantly less trust in the navigator was reported by MC's in the mHR condition, despite consistent performance. No other significant differences were observed in self-reported metrics.

The MC reported significantly greater trust in the navigator (*W* = 158, *p* = 0.01, *d* = 0.69) in the mH navigator compared to the mHR navigator (Figure 3A). However, no significant differences in trust in the navigator were observed by the SO (*p* = 0.10, Figure 3B). Additionally, self-reported team trust was not impacted for either the MC (*p* = 0.14; *M*_mH_ = 6.15 ± 0.92; *M*_mHR_ = 5.79 ± 1.41) or SO (*p* = 0.99 *M*_mH_ = 6 ± 1.31, *M*_mHR_ = 6 ± 1.05) across team configurations.

No significant differences were observed in self-reported mental workload measures by the SO (*p* = 0.38) or the MC (*p* = 0.67) across team configurations (Figures 3C, D). Performance, measured as the number of victims located, did not differ significantly (*t*(21) = 1.39, *p* = 0.18, *d* = 0.34) between mH and mHR team configurations (*M*_*mH*_ = 4.72 ± 1.31; *M*_*mHR*_ = 4.21 ± 1.38).

### 3.2 Neural signatures of taskwork

**Figure 4:**
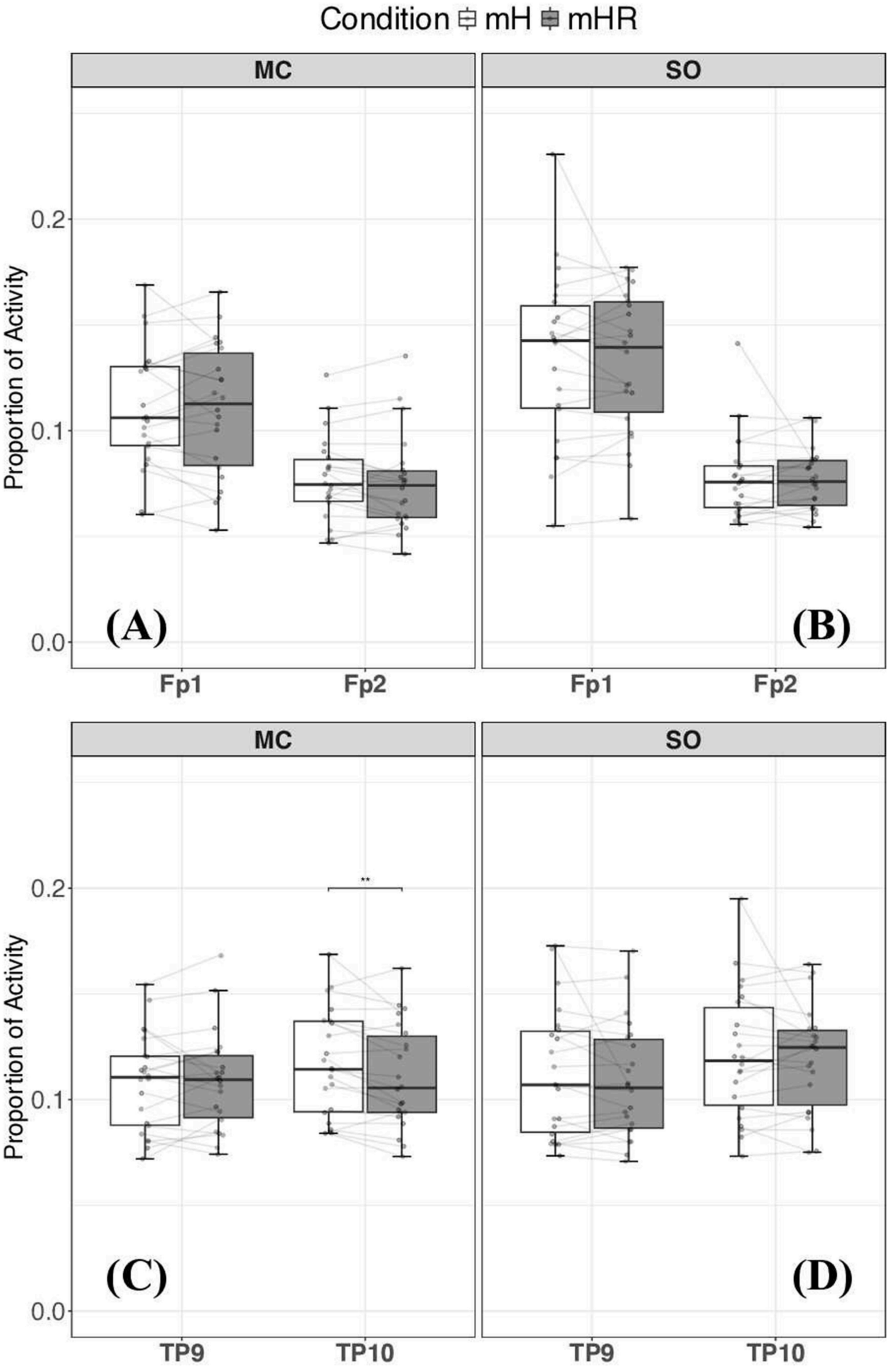
Median relative theta PSD activity at frontal **(A, B)** and temporal-parietal **(C, D)** sites for mission commanders (MC; left) and safety officers (SO; right) under Multi-Human (mH) and Multi-Human Robot (mHR) team configuration. **Represents significant differences (*p* < 0.01) between team conditions. A significant difference was observed at only the TP10 (right TPJ) for mission commanders **(C)**. No other significant differences were observed among the analyzed sites for relative theta PSD.

For MCs, the only significant team configuration difference we observed was relative theta activity at TP10, which was significantly higher (*W* = 200, *p* = 0.01, *d* = 0.56) during the mH condition than during the mHR condition. No other differences were significant between team configurations for the MCs (all *p* > 0.14) or SOs (all *p* > 0.27). Figure 4 illustrates the Theta PSD across roles and sites. Additionally, no other differences in absolute theta activity were significant between team configurations for the MCs (*M*_*mH*_ = 11.85 ± 6.31; *M*_*mHR*_ = 10.23 ± 6.84; all *p* > 0.10) or SOs (*M*_*mH*_ = 12.54 ± 5.18; *M*_*mHR*_ = 10.21 ± 4.39; all *p* > 0.18).

### 3.3 Neurodynamics of teamwork

**Figure 5:**
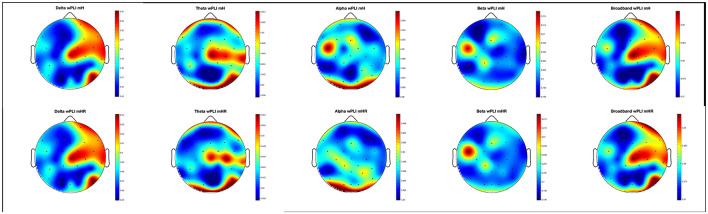
Topographic heatmaps of wPLI between Multi-Human (mH; **Top**) and Multi-Human Robot (mHR; **Bottom**) teams. wPLI is calculated per frequency band, ranging from delta **(left)** to broadband **(right)**.

Exploratory analysis on wPLI revealed significantly higher neural synchrony between MC and SO during the mHR condition than the mH condition (Figure 5) for the following sites: alpha neural synchrony at the FT9 site (*p* = 0.001, *M*_mH_ = 0.379 ± 0.259;*M*_mHR_ = 0.390 ± 0.262), beta neural synchrony at Fp1 (*p* = 0.05, *M*_mH_ = 0.196 ±, *M*_mHR_ = 0.199 ± 0.146) and T8 (*p* = 0.04, *M*_mH_ = 0.197 ± 0.147 *M*_mHR_ =0.198 ± 0.147), and delta neural synchrony at the FC2 site (*p* = 0.02, *M*_mH_ = 0.523 ± 0.312, *M*_mHR_ = 0.527 ± 0.314). Finally, significantly greater broadband synchrony was observed during mHR than the mH condition at FT9 (*p* = 0.001, *M*_mH_ = 0.373 ± 0.007, *M*_mHR_ = 0.382 ± 0.009) and O1 (*p* = 0.023, *M*_mH_ = 0.372 ± 0.010, *M*_mHR_ = 0.380 ± 0.006).

### 3.4 Mission performance prediction

**Figure 6:**
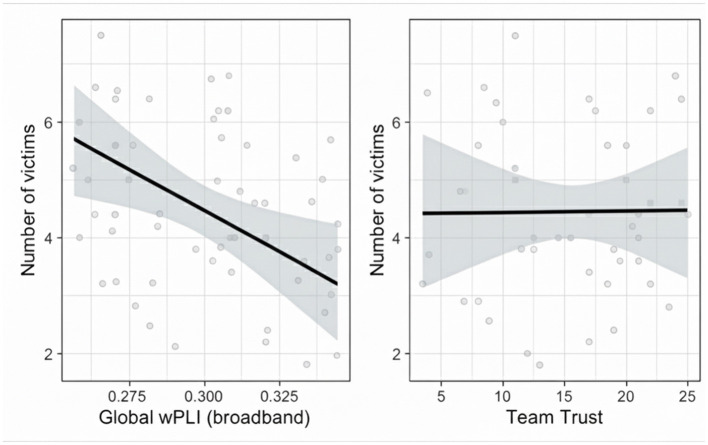
Relationship between the number of victims located and the average global broadband wPLI **(left)** and team trust ratings **(right)**. Broadband wPLI was averaged across all channels and compared to the number of victims located by each team across team configurations. Higher broadband wPLI significantly predicts fewer victims rescued, whereas subjective reports of team trust appear to have no relationship with wPLI.

The regression analysis revealed that broadband global wPLI significantly predicts team performance (*p* = 0.023, *R*^2^ = 0.18; Figure 6). Teams exhibiting higher wPLI found significantly fewer victims (β = −23.50, *p* = 0.006, *SE* = 8.14). Team trust did not significantly predict team performance (β = *0.005, p* = 0.886, *SE* = 0.004).

### 3.5 Team communication

**Figure 7:**
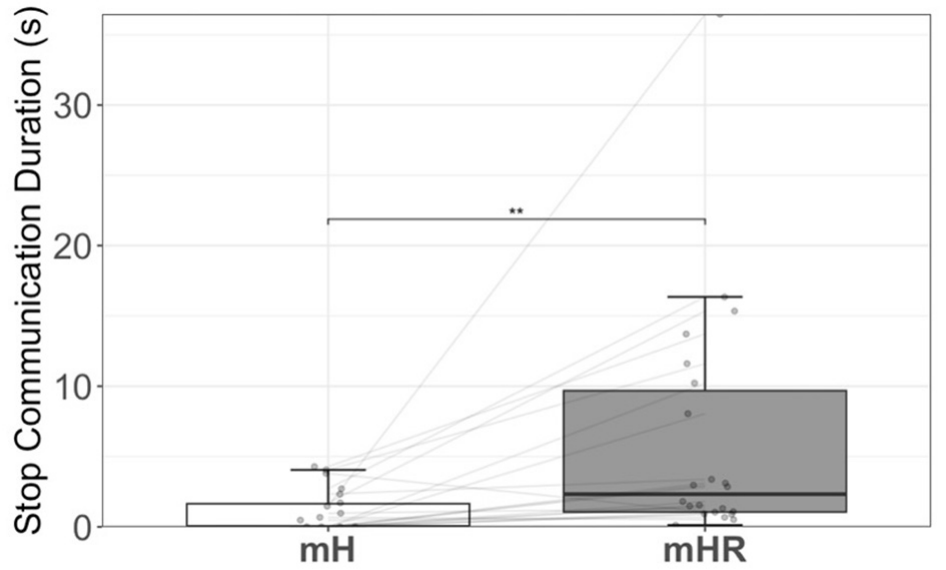
Stop communication is calculated as the amount of time the team needed to stop moving to discuss and plan their next actions. Higher stop communication durations are an indicator of less efficient searchers. We observed teams engaging in significant more stop communication during multi-human robot (mHR) trials, compared to multi-human (mH) trials.

Lastly, analysis of team communication metrics revealed significantly more stopped communication between participants during the mHR team configuration than during mH teams (*p*<*0.0*01, *M*_mH_ = 1.02*s* ± 1.47*s*, *M*_mHR_ = 6.16*s* ± 8.57*s*; Figure 7). Neither the count of MCs requesting to follow the navigator nor the hazard communication count differs significantly across team configurations (*p* > 0.11).

## 4 Discussion

This study aimed to investigate the neurocognitive dynamics of multi-human-robot teaming in SAR environments by comparing all human teams with human-robot team configurations. Key findings indicate that although robotic teammates did not reduce workload, team trust, or mission performance, they significantly altered social cognition and increased neural synchrony relative to all human teams. These results underscore the value of neurodynamic signatures in detecting subtle shifts in team interaction that subjective metrics often overlook.

### 4.1 Neurocognitive responses to taskwork

We found that neither safety officers nor mission commanders reported or showed (via neural activity) reduced mental workload when a robot teammate was introduced, thereby *disproving Hypothesis 1.1*. These findings of stable workload across team configurations and roles contrast with several industrial human-robot collaboration studies that report that robotic agents successfully reduce operator mental effort (Carissoli et al., 2024). Interestingly, no differences in mission performance, i.e., number of victims identified, were observed across team configurations. These findings challenge the “substitution myth”, i.e., the assumption that a robot with equal task performance can be seamlessly substituted for a human operator without altering the underlying cognitive architecture of the system to which they belong (Matthews and Greenspan, 2020). However, these results offer little insight into whether replacing a human teammate with a robotic agent affects team cognition.

Interestingly, our findings of lower TPJ theta PSD among mission commanders indicate that the inclusion of a robotic teammate indeed affects the social-cognitive abilities of highly involved team members (i.e., mission commanders) more than those of less involved members (i.e., safety officers). This result aligns with previous findings (Nguyen et al., 2020) and provides *partial support* for *Hypothesis* 1.2. To interpret the null findings among safety officers, we expand on a few possibilities: (1) limited engagement with the robotic system, and (2) role-specific task demands. Given the pre-determined roles established for each team, where the safety officers would focus on observing and reporting hazard, and the mission commanders would coordinate with the navigator to lead the team, we expect that the safety officers would have spent most of their time engaging with the mission commander rather than the robot, and thus, we cannot conclusively say the safety officer was perturbed by the inclusion of the robot.

The mission commander had more self-contained tasks that did not require them to engage with their teammates differently under either team configuration. Namely, the safety officer was responsible only for monitoring the danger levels in each room the team visited and reporting to the mission commander. While this task may have required participants to sustain vigilance for short periods, we do not believe the task demands for the safety officers would have a substantially different effect across conditions (Parasuraman et al., 2000). Despite this potential flaw, an interdependent team structure has been found to boost the performance of human-AI teams in related domains past that of a human or AI system alone (Vaccaro et al., 2024; Qin L. et al., 2025). While the safety officer's lack of engagement with the robot may have influenced our taskwork results, we believe this team configuration more accurately reflects the structure of an actual multi-human robot team, though future work may want to further investigate this trade-off. Analysis of self-reported trust in navigator scores provides additional support: the mission commander reported trusting a robot navigator less than a human navigator, whereas the safety officers did not change their trust levels across navigator types. While distrust is generally seen as problematic [see (Nguyen et al., 2020)], our results indicate no signs of decline in cognitive abilities, and that some additional monitoring of partner performance may enable mission commanders to remain more involved with team processes, collaboration, and joint attention (Zhang et al., 2025; Kelsen et al., 2022; Liu et al., 2018; Lachat et al., 2012). These results suggest that adding a robot to a team could alter each member's taskwork abilities, although the degree of impact may be modulated by the nature of the work. Although we observed a significant disparity in trust between human and robot navigators, a recent study found no significant differences in participants' trust behavior when interacting with AI vs. human partners who performed similarly (Kelsen et al., 2022).

### 4.2 Neural synchrony markers of teamwork

Among the sites surveyed for neural synchrony in this study, FT9 appears particularly robust, as both broadband and alpha wPLI were significantly higher during mHR trials than during mH trials. Prior research (Lachat et al., 2012) has identified the left frontotemporal site as specifically important for governing the rules of social interaction (e.g., turn-taking) and communication. Our results suggest that incorporating a robot into a dynamic team may increase collaboration among human members, indicating that mH teams are fundamentally different from mHR teams and *supporting Hypothesis 2.1*. However, these differences were not detected using subjective team trust scores, indicating that neural metrics provide valuable insight into teaming demands when robots are embedded in multiparty human teams. This result may be partially explained by our analysis of stopped communication, in which human-robot teams spent significantly more time in a stopped state. The increase in communication breakdowns could indicate that a lack of trust has manifested in behavioral detriments. Specifically, a less trusting mission commander may spend more cognitive resources monitoring the robot navigator, needing to stop more often to re-orient the team and pulling more information from the safety officer. While this behavior would not support the team's effectiveness, it could increase neural synchrony at the FT9 site without the expected benefits of increased coordination. (Short et al., 2021; Konvalinka and Roepstorff, 2012).

Given that our behavioral results suggest that mission commanders trusted the robot navigator less, these benefits may be extremely subtle, further underscoring the need for trust calibration within these teams. This conclusion was consistent with subjective ratings of team trust, which did not differ significantly across configurations, suggesting that social awareness was oriented toward collaboration rather than monitoring. The robustness of FT9 synchrony aligns with hyperscanning literature showing fronto-temporal regions as hubs for speech rhythm and turn-taking coordination (Kelsen et al., 2022). More importantly, this study prioritizes dynamic interaction, captured using neural synchrony, as the unit of analysis, as theorized within the interactive team cognition framework (Cooke et al., 2013). By doing so, the findings indicate that although team outputs (i.e., mission performance) remain comparable between mH and mHR teams, the internal processes are fundamentally transformed when a human teammate is replaced by a robotic agent.

Additionally, higher wPLI at left occipital sites during mHR configurations suggests that team members engage more in joint attention and shared visual processes while working (Cooke et al., 2013; Vicente et al., 2023). This conclusion is further supported by the observation of higher beta synchrony at left prefrontal sites, which has been linked to joint attention and collaboration in teams (Georganta and Ulfert, 2024; Czeszumski et al., 2022). The results of *Hypothesis 2.2* suggest that robot teams may impose a “hidden cost” on team members, which may not be observable using behavioral or subjective metrics but could negatively affect team outcomes over the long term. Indeed, results of our regression analysis support the idea that measures of neural synchrony are demand-oriented, as we found that more neural synchrony predicts fewer victims located. This suggests that teams may experience maladaptive neural reorganization, driven by the task and teaming demands, resulting in over-coupling or rigidity (Bassett et al., 2011). Specifically, our results suggest that in mHR team configurations, greater social cognition is required, resulting in redundant and inefficient information processing and reduced team efficiency. This negative relationship, or synchrony paradox, is supported by recent simulation research identifying negative correlations between anterior alpha synchronization and team performance, suggesting that over-coupling can indicate a maladaptive cohesive typology (Park et al., 2020). These findings deviate from traditional dyadic studies, where inter-brain synchrony is generally viewed as a positive marker of collaboration success and social facilitation.

### 4.3 Study limitations

A major limitation of this study is its exploratory nature, which focused broadly on synchrony measures. By collapsing synchrony measures across dyads into a single mean or median, we cannot evaluate how wPLI changes in response to environmental events (e.g., robot guidance). An alternative explanation for our findings is that individual team members adjust their cognitive processes in ways that inadvertently align with those of their teammates. Critically evaluating how neural synchrony changes in response to navigator directions (both human and robot) is a logical future application that may shed light on the interplay between taskwork and teamwork. Additionally, with the current setup, we are unable to map some neural metrics to behavioral outcomes. For example, it would be beneficial to pair future studies with eye-tracking to determine the direction of visual attention. Future research may consider using additional measures (e.g., eye tracking) to confirm the behavioral impact of this result, which could be interpreted as members spending more time scanning the environment for victims or monitoring the robot's behavior (Viriyopase and Narajeenron, 2025; Hergeth et al., 2016). Similarly, this study exhibited an imbalanced sex distribution across teams, which may not reflect ecologically valid teamwork. This study could have benefited from additional behavioral metrics, such as communication network analyses, which may have strengthened the utility of neural synchrony in revealing how social interactions were affected by team configuration.

Finally, the extent to which participants attributed the robot's behavior to an “autonomous” system is unknown. It is possible that some participants recognized the WoZ control and inferred that a human was controlling the robot. While the researchers aimed to reduce the likelihood that participants would attribute robot actions to human researchers, we cannot guarantee that these results are due solely to the presence of a robot. Future work should continue to investigate team neurodynamics in the presence of non-WoZ-controlled robots. Additionally, despite the robot's performance being comparable to that of a human navigator, mission commanders and safety officers may have had higher expectations of the robot, which, when unmet, led human team members to develop compensatory coordination, increasing neural synchrony.

## 5 Conclusion

The study findings provide neurodynamic evidence that both taskwork and teamwork may be fundamentally altered in multi-human-robot teams, regardless of the robots' capabilities and functions, compared with all-human teams. These neural signatures predict mission performance better than traditional teaming measures, such as team trust. Therefore, beyond dyadic interactions, multi-human robot teaming must be viewed as a fundamentally distinct team construct rather than simply an extension of human-human teaming.

## Data Availability

The raw data supporting the conclusions of this article will be made available by the authors, without undue reservation.
